# Late Recurrence of Estrogen Receptor‐Positive Breast Cancer Presenting as a Golf‐Ball‐Sized Mass in the Left Supraclavicular Fossa

**DOI:** 10.1002/ccr3.71088

**Published:** 2025-10-08

**Authors:** Tomoyo Nishi, Risa Hirata, Masaki Tago

**Affiliations:** ^1^ Department of General Medicine Saga University Hospital Saga Japan

**Keywords:** breast neoplasms, education, lymphatic metastasis, recurrence

## Abstract

A 71‐year‐old woman who, 27 years previously, had undergone left mastectomy and received postoperative hormone therapy for 1 year for breast cancer without recurrence, noticed a mass in her left supraclavicular fossa. The mass was approximately 5 cm in diameter. A biopsy confirmed the presence of metastatic breast cancer.

**Summary**

- Breast cancer can recur late, even without lymph node metastasis during diagnosis.
- Patients with a breast cancer history and a short duration of postoperative hormone therapy should be considered as having an increased risk of late breast cancer recurrence.

Estrogen‐receptor (ER)‐positive breast cancer can recur late, even without lymph node metastasis at the time of diagnosis. Recent studies have confirmed recurrence up to 32 years after the initial diagnosis [1], and a short postoperative endocrine therapy is a risk factor for recurrence. Furthermore, the shorter the duration of endocrine therapy, the higher the risk of recurrence [2]. Here, we report on a 71‐year‐old woman who underwent a left mastectomy, lymph node dissection, and postoperative hormone therapy for 1 year for ER‐positive breast cancer 27 years ago. She was followed up for 8 years postoperatively, without any recurrence. She had a body mass index of 31, indicating obesity, with no exercise habits. Seven months before presenting to our hospital, she noticed a mass in her left supraclavicular fossa that had been enlarging for 2 months. The mass became erythematous over 2 weeks, while remaining painless. The mass (diameter, approximately 5 cm) protruded from the left supraclavicular region, was firm, poorly mobile, warm to touch, non‐tender, and accompanied by erythema and vascular dilation at the apex (Figure 1). Ultrasonography revealed a > 3 cm multinodular subcutaneous mass extending from the left supraclavicular fossa to the accessory nerve region. The mass was heterogeneous and hypoechoic, with increased blood flow signals (Figure 2). Breast ultrasonography revealed no abnormalities. Contrast‐enhanced thoracoabdominal computed tomography revealed that the lymph node had infiltrated and merged with surrounding adipose tissue and muscles, with multiple small nodules suspected of metastasis observed in both lungs. Upper and lower gastrointestinal endoscopy and gynecological examinations failed to reveal the primary lesion. A biopsy of the mass performed 20 days after the initial visit showed tumor cell proliferation and mitotic figures. Immunohistochemistry revealed cytokeratin (CK)7 positivity, CK20 negativity, gross cystic disease fluid protein‐15 and GATA3 positivity, estrogen and progesterone receptor positivity, and human epidermal growth factor receptor 2 negativity, which led to the diagnosis of metastatic breast cancer. Hormone therapy was initiated on Day 34 after the initial visit, and a partial response was achieved at 8 months; by 9 months, the mass had reduced to 2.7 cm.

**FIGURE 1:**
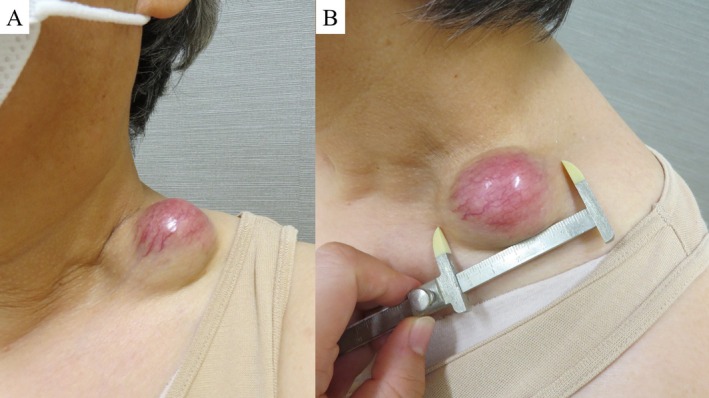
Mass in the left supraclavicular region (A: Frontal view, B: Lateral view). A protruding mass measuring 54 mm in diameter is observed in the left supraclavicular region, with a tense sensation, erythema, and capillary dilation at the apex.

**FIGURE 2:**
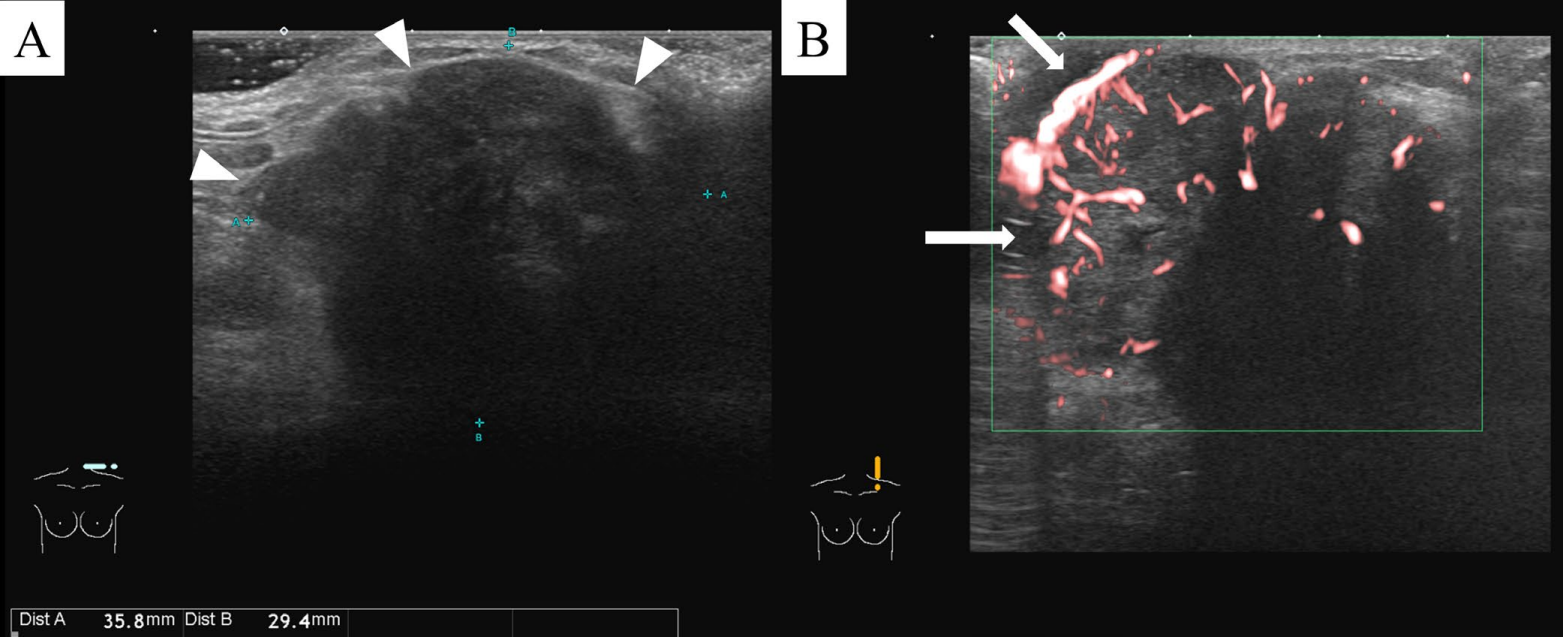
Ultrasonography images of the mass in the left supraclavicular region (A: B‐mode, B: Power Doppler mode). A hypoechoic mass with irregular margins and a lobulated, heterogeneous internal echo pattern measuring 36 × 29 mm is observed (A, arrow heads). Increased blood flow signals are observed within the mass in power Doppler mode (B, arrows).

Currently, breast cancer treatment guidelines in Japan recommend 5 years of postoperative hormone therapy [3]; however, in this case, the patient received postoperative hormone therapy for only 1 year. Patients with a history of breast cancer and a short duration of postoperative hormone therapy should be considered at an increased risk of late breast cancer recurrence.

In the present case, the patient's understanding of breast cancer recurrence risk was insufficient, and she did not suspect metastasis, leading to a 7‐month delay from noticing the mass in the supraclavicular fossa to seeking medical attention. If adequate information about late recurrence had been provided at the time of initial breast cancer diagnosis, the patient might have sought medical attention earlier.

In patients with ER‐positive breast cancer, physicians must consider the possibility of late recurrence and thoroughly inform patients that recurrence can occur, even after 30 years. Special attention is required for patients with a short duration of postoperative hormone therapy.

## Author Contributions

**Tomoyo Nishi:** conceptualization, investigation, writing – original draft. **Risa Hirata:** conceptualization, investigation, writing – original draft. **Masaki Tago:** conceptualization, supervision, writing – original draft, writing – review and editing.

## Ethics Statement

This manuscript conforms to the provisions of the Declaration of Helsinki in 1995 (as revised in Brazil 2013).

## Consent

Written informed consent was obtained from the patient for the publication of this report in accordance with the journal's patient consent policy.

## Conflicts of Interest

The authors declare no conflicts of interest.

## Data Availability

The data that support the findings of this study are available from the corresponding author upon reasonable request.
